# Social-Cognitive Predictors of Exclusive Breastfeeding among Primiparous Mothers in Addis Ababa, Ethiopia

**DOI:** 10.1371/journal.pone.0164128

**Published:** 2016-10-10

**Authors:** Anteneh Girma Minas, Makombo Ganga-Limando

**Affiliations:** 1 University of South Africa, Addis Ababa, Ethiopia; 2 University of South Africa, Collage of Human Science, Department of Health Studies, Pretoria, South Africa; Institute for Health & the Environment, UNITED STATES

## Abstract

**Background:**

Despite the presence of high impact interventions to improve infant and young child feeding, only about 52% of mothers in Ethiopia exclusively breastfeed their child for the first six months after delivery. Although the decision to breastfeed a child is ultimately that of the mother, this decision could be influenced by a variety of factors including social-cognitive ones.

**Objectives:**

The objectives of the study were to describe the breastfeeding behaviour of primiparous mothers during their prenatal period in terms of intentions/goals, outcome expectancies, self-efficacy, and socio-structural factors and assess their exclusive breastfeeding (EBF) practices as well as identify the social-cognitive predictors of EBF practices among these mothers in Addis Ababa, Ethiopia.

**Methods:**

A prospective follow up health facility-based study with quantitative methods was used with a sample of 233 primiparous women. Both structured and semi-structured questions were used for collection of data. The Statistical Package for Social Sciences (SPSS) version 21 was used for data analysis. Findings at the 95% confidence interval and P-value of 5% were reported as statistically significant.

**Results:**

39.1% (n = 59) of the respondents were found to have high breastfeeding self-efficacy, 51.4% (n = 71) have good breastfeeding outcome expectancies, and 6.5% (n = 9) respondents had supportive breastfeeding socio-structural factors. Bivariate correlation analysis showed positive and statistically significant correlation between each of breastfeeding self-efficacy, outcome expectancy, and socio-structural factors, with EBF practice. However, only breastfeeding self-efficacy and outcome expectancies were statistically significant predictors of EBF among the primiparous women when controlling for confounding variables.

**Conclusions and Recommendations:**

Health programmes aimed at improving EBF among primiparous mothers should look beyond providing health information alone. Rather improving primiparous women’s breastfeeding self-efficacy and outcome expectancy is strongly recommended. Further community based large scale research is also recommended among similar groups of women.

## Background

Exclusive breastfeeding (EBF) is globally promoted as the ideal method of infant feeding during the first six months of life due to its health benefits to both the mother and child. Among the top fifteen child survival strategies, EBF up to six months of age and breastfeeding up to 12 months are viewed as the most effective interventions with complementary feeding starting at six months as the third most effective intervention. The combination of these interventions together is estimated to prevent almost one-fifth of under-five mortality in developing countries [1]. Meanwhile, inappropriate feeding practices during the first years of life is directly and indirectly associated with more than two thirds of under-fives death due to malnutrition [2].

In Ethiopia, although almost all women breastfeed their children at some point during infancy, the practice of optimal breastfeeding is remains low. For instance, the 2011 Ethiopia Demographic and Health Survey (EDHS), found that only about 52% of mothers in Ethiopia exclusively breastfed their children for the first six months. The lowest average exclusive breastfeeding duration was reported among mothers in Addis Ababa (one month compared to the national average of 4.2 months) [3].

The decision to breastfeed a child is ultimately a choice of mothers. However, this choice is influenced by various factors including social-cognitive ones [4–6]. Thus, in order to effectively improve breastfeeding practices, interventions should be based on identified breastfeeding predicting and modifiable variables [7, 8]).

This study was part of a bigger research project by the authors (AGM and MGL) and further details of that research have been published on the African Journal of Nursing and Midwifery (AJNM). According to that publication, a positive breastfeeding attitude and early initiation of breastfeeding can predict EBF practices among primiparous women [9]. Thus, this publication gives details on the social-cognitive factors that affect EBF practice among primiparous women in Addis Ababa, Ethiopia, and supplements the previous publication evaluating the impact of social-cognitive factors on optimal breastfeeding practices.

### Statement of the Problem

Existing evidence shows that variations exist in breastfeeding practices in Ethiopia in terms of methods, duration and time of initiation. Breastfeeding practices are also associated with several factors. Social, cognitive, and environmental factors can influence a mother’s decision on breastfeeding initiation, methods and duration [7, 10–13]. Studies evaluating these aspects are well documented in Ethiopia and elsewhere, but the strength of their combined influence on the early initiation and duration of exclusive breastfeeding has yet to be fully established.

Primiparous women on the contrary needs to get more attention during social and behaviour change communication interventions as they lack prior experiences regarding breastfeeding. Thus, determining the socio-cognitive predictors of breastfeeding practices among this particular group of women will assist public health professionals and health authorities to refocus breastfeeding promotion strategies and interventions. Consequently, it will lead to the improvement of the child survival and to effective management of limited resources for the public health services.

### Conceptual Framework

Review of literatures showed that various factors, such as child and maternal health conditions [14], behavioural and psychosocial [15], socio-structural [16] and social-cognitive [17], affect breastfeeding practices. Therefore, breastfeeding behaviour can be viewed as a product of multiple factors.

According to McAlister, Perry, and Parcel [18],unlike most behavioural and social theories which focus on individual, social, and environmental factors that determine individual or group behaviour, social cognitive theory (SCT) postulates that human behaviour is the product of the dynamic interplay of personal, behavioural, and environmental influences [18]. Accordingly, the researcher used Bandura’s SCT as a conceptual framework for this study [19].

**Fig 1:**
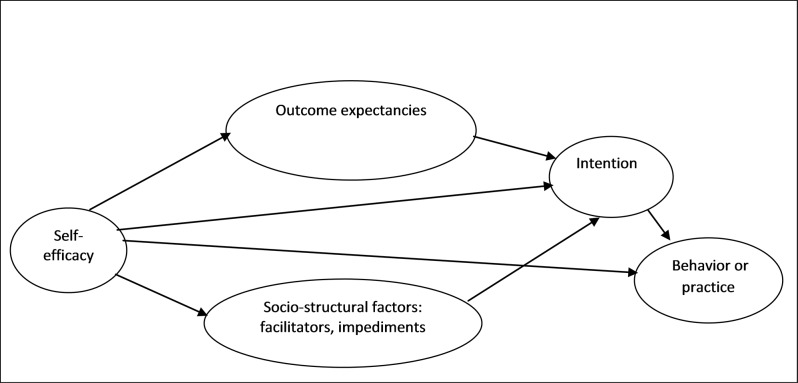
Social Cognitive Theory.

Originally, SCT stressed that behaviour is determined by the interaction of three main factors: goals/intentions, self-efficacy, and outcome expectancies [20]. However, over the last decade, social-structural factors were added to the original triad of the SCT [19]as depicted in Fig 1.

In SCT, goals/intentions are the plans to act or intent to perform a behaviour, while outcome expectancies refer to the values attached to the anticipated results of a person’s behaviour. Self-efficacy is the individual’s beliefs in his/her capabilities to successfully perform a behaviour, which is usually assessed as the degree of confidence that an individual has that she/he could still perform the behaviour in the face of various influences. The socio-structural factors in SCT refer to factors that facilitate or inhibit the execution of behaviour. With respect to health behaviour, these factors may include living conditions, health, political, economic, or environmental systems [19, 21].

In this study, all the four components (goals/intentions, outcome expectancies, self-efficacy, and socio-structural factors) were used in order to identify the social cognitive predictors of EBF practices among primiparous women. Fig 1 depicts the SCT model.

## Objectives

The specific objectives of this study were to determine the social-cognitive predictors of EBF practices among primiparous mothers in Addis Ababa, Ethiopia.

## Methodology

As described above, this study was part a bigger research project by the authors (AGM and MGL) and further details of the study methodology have been published elsewhere [9].

This study took place in Addis Ababa using a hospital-based follow up study design with quantitative methods and data were collected during April 2014 to February 2015. Primiparous women at their last trimester of pregnancy were study units, and the sample size of the study was 233. Among the six public hospitals providing maternity care in the study area, one hospital was purposively selected for this study based on its higher client caseload. The selection of this study hospital was to ensure access to an adequate sample size and maintain a minimum duration of data collection given the cost and time implications. Being a primiparous women, and at the third trimester of pregnancy, and attending antenatal care at the selected hospital were the inclusion criteria, while being referred to the study hospital from other health facilities due to medical complications, and developing medical complications during delivery were the exclusion criteria.

Open and closed ended questionnaires for the data collection were adopted from various previous studies on breastfeeding, and were piloted to check if they could be understood by the data collectors and the study participants. Five experienced nurses administered these questionnaires and collected the data in three phases

The research was approved by the ethical review committee of the University of South Africa (UNISA) and the Addis Ababa City Administration Health Bureau (AACAHB). Permission to conduct the study was also obtained from the study hospital. The consent form was also approved by these agencies. Written informed consent was obtained from study participants at each phase of the data collection processes. Confidentiality of participants was maintained as only the researcher has access to the data.

The dependent variable was EBF practices, while independent variables were breastfeeding self-efficacy, intentions, outcome expectancies, and social-structural factors. Operational definition of these variables are:

- *Breastfeeding self-efficacy* is the primiparous mother’s belief and confidence in her ability to exclusively breastfeed her child for the first six months after delivery. This was measured using a five point Likert scale, and ‘strongly agree’ or ‘agree’ responses to the correct statements, and ‘strongly disagree’ or ‘disagree’ responses to the negative statements were computed together to classify the study participants as having high breastfeeding self-efficacy. Meanwhile, the converse responses were computed to classify them as having low breastfeeding self-efficacy.
- *Breastfeeding intentions/goals* are a primiparous mother’s intentions regarding breastfeeding options and duration. This was measured using open ended questions that directly ask the intended methods and duration of breastfeeding.
- *Breastfeeding outcome expectancies* are what a primiparous mother believes and expects from exclusively breastfeeding her child for six months. This was measured using a five point Likert scale, and ‘strongly agree’ or ‘agree’ responses to the correct statements, and ‘strongly disagree’ or ‘disagree’ responses to the incorrect statements were computed together to classify the study participants as having high breastfeeding outcome expectancy. On contrary, the other responses were computed to classify them as having low breastfeeding outcome expectancy.
- *Socio-structural factors* are social and environmental factors that positively or negatively affect primiparous mothers’ breastfeeding practices. These were also measured using a Likert scale of five points, ‘strongly agree’ or ‘agree’ responses to the correct statements, and ‘strongly disagree’ or ‘disagree’ responses to the incorrect statements were computed together to classify the study participants as having supportive breastfeeding socio-structural factors. However, the other responses were computed to classify them as having not-supportive breastfeeding socio-structural factors.
- *Social-cognitive predictors* are behavioural, personal, social, and environmental factors that predict the practice of breastfeeding among primiparous mothers.

The common socio-demographic variables such as age, level of education, employment status, and family income were also considered.

Statistical Package for Social Science (SPSS) version 21 was used for data entry and analysis. Descriptive and inferential statistics, mainly frequency distributions, chi-square tests, correlation analysis and multiple logistics regression were employed. The Spearman Rank Order (Spearman rho) correlation was used to explore the strength and direction of relationship as well as the level of significance regarding correlation between breastfeeding intentions, self-efficacy, outcome expectancy and social structural factors with EBF practices. Findings at the 95% confidence interval (CI) and p-value of 5% were reported as statistically significant.

## Results

### Socio-demographic characteristics

As described previously, the study participants’ ages ranged from 17 to 40 years, about 46.1% (n = 65) were not employed, and about 72.3% (n = 102) of the respondents had secondary and post-secondary education [9].

### Breastfeeding intentions

Of the respondents, 97.9% (n = 138) of them had intended to breastfeed their child after delivery. Of these groups of mothers about 51.4% (n = 71) of them had intended to exclusively breastfeed their child for six months, while the remaining 48.6% (n = 67) respondents had intended mixed feeding.

### Breastfeeding self-efficacy

**Fig 2:**
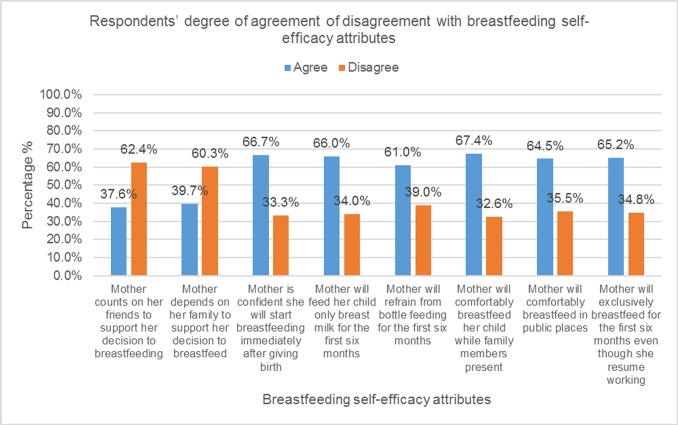
Respondents degree of agreement and disagreement with breastfeeding self-efficacy attributes.

Respondents breastfeeding self-efficacy assessment showed that most of the respondents were self- confident against the attributes of breastfeeding self-efficacy. Finding on the breastfeeding self-efficacy attributes are depicted in Fig 2.

Meanwhile, study participants’ overall breastfeeding self-efficacy assessment showed that, of those who had intended to breastfeed their children after delivery, about 40.4% (n = 57) of study participants had high breastfeeding self-efficacy (i.e. self-confidence), while the other 59.6% (n = 84) had low breastfeeding self-efficacy.

### Breastfeeding outcome expectancy

Similarly, breastfeeding outcome expectancy assessment showed that most of the respondents had high breastfeeding outcome expectancy. Table 1 describes finding on study participants’ breastfeeding outcome expectancies.

**Table 1:** Description of respondents’ degree of agreement or disagreement with breastfeeding outcome expectancy attributes (N = 141).

Respondents’ degree of agreement of disagreement with…	Totally agree	Agree	Neither/Nor	Disagree	Totally disagree
Mother thinks colostrum protects her child from illnesses or infections	73 (51.8%)	42 (29.8%)	13 (9.2%)	8 (5.7%)	5 (3.5%)
Mother thinks early initiation of breastfeeding is good for her own health	54 (38.3%)	51 (36.2%)	21 (14.9%)	9 (6.4%)	6 (4.3%)
Mother thinks exclusive breastfeeding is enough for the nutritional requirements of her child for the first six months	72 (51.1%)	48 (34%)	4 (2.8%)	10 (7.1%)	7 (5%)
Mother thinks she can save money if she exclusively breastfeeding her child for six months	41 (29.1%)	61 (43.3%)	5 (3.5%)	26 (18.4%)	8 (5.7%)
Mother thinks her child will feel more comfortable if she breastfeeds him/her	74 (52.5%)	43 (30.5%)	6 (4.3%)	11 (7.8%)	7 (5.0%)
Mother thinks denying additional liquids/foods in the first 6 months will benefit her child	68 (48.2%)	49 (34.8%)	6 (4.3%)	14 (9.9%)	4 (2.8%)

Similarly, the assessment of the study participants’ overall breastfeeding outcome expectancies showed that of those who intended to breastfeed their child, 52.5% (n = 74) had high/good breastfeeding outcome expectancies, while 47.5% (n = 67) of respondents had less/poor breastfeeding outcome expectancies.

### Breastfeeding socio-structural factors

Similarly, study participants’ overall socio-structural factors assessment showed that of the respondents who had intended to breastfeed, about 6.4% (n = 9) of them had supportive breastfeeding socio-structural factors, while a majority of the respondents (93.6%, n = 102) did not have supportive breastfeeding socio-structural factors.

### Breastfeeding practices

As described previously, 64.3% (n = 90) of the study participants initiated breastfeeding within an hour of delivery and 34.3% (n = 48) of the study participants were still exclusively breastfeeding by the fifth month after delivery [9].

### Association between breastfeeding social cognitive constructs and EBF

There was a small and positive correlation between the respondents’ intention to breastfeed and their EBF practice (rho = 0.101), but the correlation was not statistically significant (P-value = 0.235). There was small and positive correlation between study participants’ intention to EBF and EBF practices (rho = 0.059), but the correlation was not statistically significant (P-value = 0.487). However, correlation between respondents’ breastfeeding self-efficacy and EBF practice by at least the fifth month post-partum showed a high, positive and statistically significant correlation between the two variables (rho = 0.504, P-value < 0.01). Table 2 presents the correlation analysis between study participants’ breastfeeding self-efficacy and EBF practice by the fifth month after delivery.

**Table 2:** Correlations between breastfeeding self-efficacy and EBF practices (N = 140).

			Breastfeeding self-efficacy	EBF at least by at least fifth month
Spearman's rho	Breastfeeding self-efficacy	Correlation Coefficient	1.000	0.504**
		P-value	.	0.000
	EBF at least by at least fifth month	Correlation Coefficient	0.504**	1.000
		P-value	0.000	.

Note: **Correlation is significant at the 0.01 level (2-tailed).

The correlation between respondents breastfeeding outcome expectancy and their EBF practice was medium, positive, and statistically significant (rho = 0.381, P-value < 0.01). Table 3 presents the correlation analysis between study participants’ breastfeeding outcome expectancy and EBF practice by the fifth month after delivery.

**Table 3:** Correlations between respondents breastfeeding outcome expectancy and EBF (N = 140).

			Breastfeeding outcome expectancy	EBF at least by fifth month
Spearman's rho	Breastfeeding outcome expectancy	Correlation Coefficient	1.000	0.381**
		P-value	.	0.000
	EBF at least by fifth month	Correlation Coefficient	0.381**	1.000
		P-value	0.000	.

Note: **Correlation is significant at the 0.01 level (2-tailed).

There was a medium, positive and statistically significant correlation between respondents’ breastfeeding socio-structural factors and EBF by at least the fifth month after delivery (rho = 0.240, P-value < 0.01). Table 4 presents the correlation between socio structural factors and EBF.

**Table 4:** Correlations between study participants’ socio-structural factors and EBF (N = 140).

			Breastfeeding socio structural factors	EBF at least by the fifth month
Spearman's rho	Breastfeeding socio structural factors	Correlation Coefficient	1.000	0.240**
		P-value	.	0.004
	EBF at least by fifth month	Correlation Coefficient	0.240**	1.000
		P-value	0.004	.

Note: **Correlation is significant at the 0.01 level (2-tailed).

### Social-cognitive predictors of exclusive breastfeeding

Bivariate correlation analysis between each of the socio-cognitive constructs and EBF practice showed that there were positive and statistically significant correlations between breastfeeding self-efficacy, outcome expectancy, and socio-structural factors with EBF practice.

There was a statistically significant positive, small correlation between exclusive early initiation of breastfeeding and EBF by at least the fifth month (rho = 0.224, p-value < 0.01). Table 5. Presents correlation analysis between exclusive early initiation of breastfeeding and EBF.

**Table 5:** Correlations between exclusive early initiation of breastfeeding and exclusive breastfeeding (n = 140).

			Exclusive early initiation of breastfeeding	EBF at least by the fifth month
Spearman's rho	Exclusive early initiation of breastfeeding	Correlation Coefficient	1.000	0.224**
		P-value	.	0.008
	EBF at least by the fifth month	Correlation Coefficient	0.224**	1.000
		P- value	0.008	.

Note: **Correlation is significant at the 0.01 level (2-tailed).

Multiple logistic regression analysis showed that only two of these independent variables made a statistically significant contribution to this model, and these were breastfeeding self-efficacy ((x^2^(1df) = 1.990 (p-value <0.01)) and breastfeeding outcome expectancy ((x^2^(1df) = 1.420 (p-value <0.01)). Table 6 presents the results of the multiple logistics regression for predictors of EBF.

**Table 6:** Multiple logistic regression predicting EBF (N = 140).

	B value	S.E.	Wald	Degree of freedom	P-value	Adjusted Odds Ratio (AOR)	95% C.I. AOR	
							Lower	Upper
Breastfeeding self-efficacy	1.990	.496	16.089	1	0.000	7.319	2.767	19.357
Breastfeeding outcome expectancy	1.420	.505	7.907	1	0.005	4.136	1.537	11.127
Breastfeeding socio-structural factors	1.562	.990	2.491	1	0.115	4.770	.685	33.201
Exclusive early initiation of breastfeeding	0.409	.576	0.504	1	0.478	1.505	.487	4.650
Constant	4.924	.971	25.731	1	.000	.007		

The combined strength of two social-cognitive predictors of EBF (*breastfeeding self-efficacy and breastfeeding outcome expectancy*) was also assessed. Accordingly, the correlation between the combined breastfeeding self-efficacy and breastfeeding outcome expectancy with EBF was high, positive and statistically significant, as well as stronger than the correlation that each of the breastfeeding socio-cognitive constructs have independently with EBF practices (rho = 0.558, P-value <0.01). Table 7 presents the correlation analysis between combined breastfeeding self-efficacy and outcome expectancy with EBF practices.

**Table 7:** Correlations between combined breastfeeding self-efficacy and breastfeeding outcome expectancy with EBF (N = 140).

			Combined breastfeeding self-efficacy and breastfeeding outcome expectancy	EBF at least by fifth month
Spearman's rho	Combined breastfeeding self-efficacy and breastfeeding outcome expectancy	Correlation Coefficient	1.000	0.558**
		P-value	.	0.000
	EBF at least by fifth month	Correlation Coefficient	0.558**	1.000
		P-value	0.000	.

Note: **Correlation is significant at the 0.01 level (2-tailed).

## Discussion

This study identified several social-cognitive predictors of EBF practices among primiparous women, including breastfeeding self-efficacy, breastfeeding outcome expectancies, breastfeeding intention, and breastfeeding socio-structural factors.

Breastfeeding self-efficacy was found to be the strongest predictor of EBF with an adjusted odds ratio of 7.32 (95% C.I.: 2.77, 19.36). This indicated that mothers with high breastfeeding self-efficacy were over seven times more likely to exclusively breastfeed their child for at least the first five months after delivery than those with low breastfeeding self-efficacy, controlling for all other factors. This means that mothers with good breastfeeding self-efficacy tend to practice EBF compared with those with low breastfeeding self-efficacy. Glassman, McKearney, Saslaw, and Sirota also found that higher breastfeeding self-efficacy scores were associated with more breastfeeding and were associated with EBF, and they concluded that breastfeeding self-efficacy was the sole, modifiable factor associated with EBF [22]. Thus, it can be concluded that interventions aimed at improving EBF practices among primiparous women should consider improving the breastfeeding self-efficacy of mothers. Authors Wu, Hu, McCoy and Efird also recommend that interventions that aim to increase breastfeeding practices, should also consider improving maternal breastfeeding self-efficacy [23].

Breastfeeding outcome expectancy was also found to be strong predictor of EBF among primiparous women with an adjusted odds ratio of 4.14 (95% C.I.: 1.54, 11.13), indicating that mothers with high breastfeeding outcome expectancies were over four times more likely to exclusively breastfeed their infants compared with mothers having low breastfeeding outcome expectancies. This finding demonstrates that respondents with good breastfeeding outcome expectancies are more likely to exclusively breastfeed when compared to those with low breastfeeding outcome expectancies.

Most of the study participants had inadequate socio-structural factors to support them with EBF, despite the importance of having supportive socio-structural factors in the promotion of optimal breastfeeding practices. *Studies conducted elsewhere recommend supportive socio-structural factors for promotion of EBF practices*. For instance authors Ishak, Adzan, Quan, Shafie, Rani, and Ramli concluded that in ensuring a successful breastfeeding practice, apart from improving the knowledge and attitude of mothers, issues surrounding culture and traditions as well as improving delivery of readily available support should be addressed [24]. However, the correlation of socio structural factors with EBF practices was not statistically significant when controlling for other confounding factors.

Similarly, exclusive early initiation of breastfeeding practice was a statistically significant predictor of EBF by at least the fifth month (rho = 0.224, p-value < 0.01) in bi-variate analysis. This finding indicates that mothers who gave only breastmilk to their infants in the first hour after delivery were more likely to exclusively breastfeed for the first five months compared to those who gave additional liquids/foods to their child after delivery.

Meanwhile, only breastfeeding self-efficacy and breastfeeding outcome expectancy were identified as statistically significant predictors of EBF by the fifth month after delivery. The strength of correlation was even stronger when these two social-cognitive predictors were combined, indicating that mothers with both high breastfeeding self-efficacy and high breastfeeding outcome expectancies tend to practice more EBF by at least the fifth month as compared to mothers with either high breastfeeding self-efficacy or breastfeeding outcome expectancy.

## Conclusion and Recommendations

This study analysed and identified the social-cognitive predictors of EBF practices among primiparous mothers in Addis Ababa, Ethiopia. Accordingly, breastfeeding self-efficacy and breastfeeding outcome expectancies were statistically significant predictors of EBF.

Health programmes aimed at optimizing EBF among primiparous women should look beyond providing health information alone. Rather, improving their breastfeeding self-efficacy and outcome expectancies is essential.

Improving primiparous women’s breastfeeding self-efficacy could be achieved during group and individualized health education sessions either during community or health facility based services. Girls’ and women’s empowerment programmes could contribute to such initiatives.

Breastfeeding outcome expectancies are another important factor that should be considered in the promotion of EBF practices. Thus, social and behaviour change communication interventions focusing on EBF should emphasize giving complete and clear information, as well as the expected benefits of EBF for the child, mother, family and the community at large. Above all, EBF social and behaviour change communication interventions aimed at addressing both breastfeeding self-efficacy and breastfeeding outcome expectancies could benefit more than interventions aimed at addressing the individual constructs alone.

Further community based large scale qualitative research is also strongly recommended.

## Limitations of the Study

The limitations of this study were already reported elsewhere, and issue of lost to follow up and social desirability biases could not be ruled out [9].

## Supporting Information

S1 File This is the questionnaire for stage one of the data collection. (DOC)

S2 File This is the questionnaire for stage two of the data collection. (DOC)

S3 File This is the questionnaire for stage three of the data collection. (DOC)
