# Supplementary material for: Social-Cognitive Predictors of Exclusive Breastfeeding among Primiparous Mothers in Addis Ababa, Ethiopia
Source: PLoS One. 2016 Oct 10;11(10):e0164128. doi: 10.1371/journal.pone.0164128 (PMC5056706; doi:10.1371/journal.pone.0164128)
Supplement: S1 File — (DOC) [file pone.0164128.s001.doc]

Identification

| Name |  | |
| --- | --- | --- |
| Medical Record Number |  | |
| ANC Registration Number |  | |
| Sample Code |  | |
| Is the mother interviewed at her last term? | Yes  No | If “**Yes**” , Date of interview in Ethiopian calendar___________ |

| **SECTION I: SOCIO-DEMOGRAPHIC CHARACTERISTICS** | | | |
| --- | --- | --- | --- |
| SN | Questions | Responses | Skip |
|  | How old are you? | ______________ years |  |
|  | What is your religion? | Orthodox Christian  Catholic Christian  Protestant Christian  Muslim  Other (specify) ________ |  |
|  | What is your educational background? | No formal education  Primary level  Secondary level  Post-secondary level |  |
|  | Where do you live currently? | Addis Ababa  Outside Addis Ababa |  |
|  | What is your marital status? | Single  Married  Separated  Widowed | 107  107 |
|  | If “***married*** or ***single***” are you currently living with your spouse or partner? | Yes  No |  |
|  | What is your employment status | Not employed  Own business (self-employed)  Employed (full time, or per time) | 110  110 |
|  | If “***employed to Q107***”, will your employer give you maternity leave? | Yes  No | 110 |
|  | If “***Yes to Q108***”,   1. How long is your maternity leave? | ----------- (months) |  |
|  | 1. Will your employer pay you during your maternity leave? | Yes  No |  |
|  | How much is your family’s income per month? | __________Birr |  |

| **SECTION IV: BREASTFEEDING INTENTIONS** | | | | | |
| --- | --- | --- | --- | --- | --- |
| **SN** | **Questions** | **Responses** | | | **Skip** |
|  | Do you intend to breastfeed your child? | Yes  No  Not decided yet | | |  |
|  | What motivates your answer to **Q201**? |  | | | |
|  | If “**Not decided yet to Q201**”, when do you think you will decide whether you breastfeed you child? | Before giving birth  After giving birth  I don’t know | | | |
|  | If “**Yes to Q201**”, how soon will you initiate breastfeeding after delivery? | __________ minutes or  __________ hours | | |  |
|  | What motivates you initiate breastfeeding with in the above duration? |  | | |  |
|  | If “Y**es to Q201**”, for how long are you planning to breastfeed your child? | ___________ months | | |  |
|  | Why do you intend to breastfeed your child for the above duration? |  | | |  |
|  | If “Y**es to Q201**”,   1. What additional things besides to your breast milk will you feed your child for the first six months? | | | |  |
|  |  | **Yes** | **No** | **Not decided yet** |  |
| A1 | Nothing |  |  |  |  |
| A2 | vitamin drops / medicines as drop |  |  |  |  |
| A3 | ORS |  |  |  |  |
| A4 | Plain water |  |  |  |  |
| A5 | Infant formula |  |  |  |  |
| A6 | Milk (tinned, powdered, or fresh animal milk) |  |  |  |  |
| A7 | Clear broth (vegetable soup) |  |  |  |  |
| A8 | Juice or juice drinks |  |  |  |  |
| A9 | Sour milk or yoghurt |  |  |  |  |
| A10 | Thin porridge |  |  |  |  |
| A11 | Other (specify)_______ |  |  |  |  |
|  | 1. Why do you intend (*choose from the above response*) to feed your child? |  | | | |

| **SECTION V**: **BREASTFEEDING SELF-EFFICACY** | | | | | | |
| --- | --- | --- | --- | --- | --- | --- |
| SN | Questions | Response | | | | |
|  | Please tell me how strongly you agree or disagree with each of the following statements. Do you totally agree (1), agree (2), neither agree nor disagree (3), disagree (4) or totally disagree (5) with….. | | | | | |
|  |  | 1 | 2 | 3 | 4 | 5 |
| a | I can count on my friends to support my decision to breastfeed |  |  |  |  |  |
| b | I will depend on my family to support my decision to breastfeed |  |  |  |  |  |
| c | I am confident that I will start breastfeeding immediately after I gave birth |  |  |  |  |  |
| d | I will feed my child only breast milk for the first six months |  |  |  |  |  |
| e | I will refrain from bottle feeding for the first 6 months |  |  |  |  |  |
| f | I will comfortably breastfeed my child with my family members present |  |  |  |  |  |
| g | I will comfortably breastfeed in public places |  |  |  |  |  |
| h | I will breastfeeding exclusively for six months even though I resume working |  |  |  |  |  |

| **SECTION VI: BREASTFEEDING OUTCOME EXPECTANCY** | | | | | | |
| --- | --- | --- | --- | --- | --- | --- |
| SN | Questions | Responses | | | | |
|  | Please tell me how strongly you agree or disagree with each of the following statements. Do you totally agree (1), agree (2), neither agree nor disagree (3), disagree (4) or totally disagree (5) with….. | | | | | |
|  |  | 1 | 2 | 3 | 4 | 5 |
|  | I think that colostrum protects my child from illnesses or infections. |  |  |  |  |  |
|  | I think that my early initiation of breastfeeding will help me in reducing excessive bleeding after delivery. |  |  |  |  |  |
|  | I think that breast milk during the first six months contains the nutrient my child requires. |  |  |  |  |  |
|  | I think that I can save money, if I exclusively breastfeed my child during the first six months. |  |  |  |  |  |
|  | I think that my breastfeeding will strengthen the bond between my child and myself. |  |  |  |  |  |
|  | I think that denying additional liquids and/or foods during the first six months will benefit my child. |  |  |  |  |  |

| **SECTION VII: SOCIO-STRUCTURAL FACTORS** | | | | | | | | | | | | |
| --- | --- | --- | --- | --- | --- | --- | --- | --- | --- | --- | --- | --- |
| SN | Questions | Responses | | | | | | | | | Skip | |
|  | Is this your first antenatal care? | Yes  No | | | | | | | | | 503 | |
|  | If “**No to Q701**”, how many antenatal cares have you received so far including this one? | _________________ | | | | | | | | |  | |
|  | Have you received health education on breastfeeding during the antenatal visits? | Yes  No | | | | | | | | | 505 | |
|  | If “**Yes to Q703**”,   1. How much are you satisfied with the health education you received? | Very satisfied  Satisfied  Neither /nor  Not satisfied  Not satisfied at all | | | | | | | | |  | |
|  | 1. Will you recommend to other women the health education sessions on breastfeeding in the health facility you have been visiting for antenatal care? | Yes  No | | | | | | | | |  | |
|  | Please tell me how strongly you agree or disagree with each of the following statements. Do you totally agree (1), agree (2), neither agree nor disagree (3), disagree (4) or totally disagree (5) with….. | | | | | | | | | | | |
|  |  | | | | 1 | | 2 | | 3 | | 4 | 5 |
| a | I have had access to health services during the last five to six months in a nearest public health facility. | | | |  | |  | |  | |  |  |
| b | My family supported my visit to the health facilities for antenatal care | | | |  | |  | |  | |  |  |
| c | Health care providers have given me adequate information about breastfeeding during the last five to six months. | | | |  | |  | |  | |  |  |
| d | My spouse or partner will support me to raise my child. | | | |  | |  | |  | |  |  |
| e | I am confident my employer (or business partner) will support me that I will be able to breastfeed my child. | | | |  | |  | |  | |  |  |
| f | Breast milk substitute marketers will have lesser influence in my decision to buy formula milk for my child during the first six months after birth. | | | |  | |  | |  | |  |  |
| g | I am confident that I can join community activities (for example, festivities, religious or other activities) in the same way as anyone else can. | | | |  | |  | |  | |  |  |
| h | My existing health problems can affect my breastfeeding practice. | | | |  | |  | |  | |  |  |
|  | | | | | | | | | | | | |
|  | Who is sharing home with you? | | No one,  Siblings,  Parents,  Grandparents,  Close friend,  Others (Specify) ______ | | | | | |  | | | |
|  | How many of your siblings (your brothers, sisters cousins…) were breastfed exclusively for six months? | | All of them  Some of them  None of them  Don’t know | | | | | |  | | | |
|  | Please tell me how strongly you agree or disagree with each of the following statements. Do you totally agree (1), agree (2), neither agree nor disagree (3), disagree (4) or totally disagree (5) with….. | | | | | | | | | | | |
|  |  | | | 1 | | 2 | | 3 | | 4 | | 5 |
| a | My close relatives’ attitude towards my intentions to breastfeed my child is positive. | | |  | |  | |  | |  | |  |
| b | My close friends’ attitude towards my intentions to breastfeed my child is positive. | | |  | |  | |  | |  | |  |
| c | My spouse’s/partner’s attitude towards my intentions to breastfeed my child is positive. | | |  | |  | | ` | |  | |  |

The questionnaire ends here. Would you like to ask me any further questions?

Thank you very much for your time and information.
