# Supplementary material for: Social-Cognitive Predictors of Exclusive Breastfeeding among Primiparous Mothers in Addis Ababa, Ethiopia
Source: PLoS One. 2016 Oct 10;11(10):e0164128. doi: 10.1371/journal.pone.0164128 (PMC5056706; doi:10.1371/journal.pone.0164128)
Supplement: S2 File — (DOC) [file pone.0164128.s002.doc]

**Section I: Identification**

| Mother’s name |  |
| --- | --- |
| Sample Code |  |
| Is the mother still willing to be interviewed? | Yes  No |
| How long have been since the mother give birth? | _______hours or ________ days |
| Place of delivery | Home,  Health facility |
| Mode of delivery | Vaginal delivery  Caesarean section |
| What was the birth outcome?  ***(End the interview if the birth outcome is note Live birth)*** | Live birth  Still birth  New born died immediately  Mother died immediately |
| What is the sex of the new-born? | Male  Female  Twins; male and female  Twines; both male  Twines; both females |
| Date of interview |  |
| Interviewer’s name and signature |  |

**Section II: Early Breastfeeding practices (0-1hour)**

| **SN** | **Questions** | | **Responses** | | **Skip** |
| --- | --- | --- | --- | --- | --- |
|  | Did you start breastfeeding your child within one hour after giving birth? | | Yes  No | | 604 |
|  | If “**No to #601”,**   - Could it be due to the following reasons? | | | | |
|  |  | | | Yes | No |
|  | | I have no adequate breast milk | |  |  |
|  | | I believe the new-born is not ready | |  |  |
|  | | I do not want to breastfeed | |  |  |
|  | | I gave birth by caesarean section | |  |  |
|  | | I have medical conditions that prevented me from breastfeeding | |  |  |
|  | | Other (specify)_______________ | |  |  |
|  | If “**No to #601**”,   - How soon did you initiate breastfeeding after you give birth | | | within ____ hours | |
|  | Did you give ____________ *(choose from the following options)* for your child within one hour after you give birth? | | | | |
|  |  | | | Yes | No |
|  | | vitamin/medicines as drop | |  |  |
|  | | ORS | |  |  |
|  | | Plain water | |  |  |
|  | | Infant formula | |  |  |
|  | | Milk (tinned, powdered, or fresh animal milk) | |  |  |
|  | | Clear broth (vegetable soup) | |  |  |
|  | | Juice or juice drinks | |  |  |
|  | | Sour milk or yoghurt | |  |  |
|  | | Thin porridge | |  |  |
|  | | Other (specify)_____________ | |  |  |

I have finished my questions. Would you like to ask me any questions?

Thank you a lot for your time and information
