# Supplementary material for: Social-Cognitive Predictors of Exclusive Breastfeeding among Primiparous Mothers in Addis Ababa, Ethiopia
Source: PLoS One. 2016 Oct 10;11(10):e0164128. doi: 10.1371/journal.pone.0164128 (PMC5056706; doi:10.1371/journal.pone.0164128)
Supplement: S3 File — (DOC) [file pone.0164128.s003.doc]

**Section I: Identification**

| Mother’s name |  |
| --- | --- |
| Sample Code |  |
| Is the mother still willing to be interviewed? | Yes No |
| How long have been since the mother give birth? | _______months |
| Is the mother eligible to be interviewed? | Yes No |
| Date of interview in Ethiopian calendar |  |
| Interviewer’s name and signature |  |

**Section II: Breastfeeding Practices (5-7months of age)**

| **SN** | **Questions** | | **Responses** | |
| --- | --- | --- | --- | --- |
|  | In the last 24hours, did you give ____________ to your child? | | | |
|  |  | | **Yes** | **No** |
|  | | Breast milk |  |  |
|  | | Vitamin/medicines as drop |  |  |
|  | | ORS |  |  |
|  | | Plain water |  |  |
|  | | Infant formula |  |  |
|  | | Milk (tinned, powdered, or fresh animal milk) |  |  |
|  | | Clear broth (vegetable soup) |  |  |
|  | | Juice or juice drinks |  |  |
|  | | Yoghurt |  |  |
|  | | Thin porridge |  |  |
|  | | Other (specify)______________ |  |  |
|  | If ‘**the mother gives additional food or liquids**’, ask   - Your reason to give ……was…? | | | |
|  |  | | **Yes** | **No** |
|  | | Low milk supply |  |  |
|  | | The child not being satisfied |  |  |
|  | | Mothers resume working |  |  |
|  | | Breastfeeding was painful |  |  |
|  | | Breastfeeding was time consuming |  |  |
|  | | Physicians recommendation |  |  |
|  | | Other (specify)______________ |  |  |

I have finished my questions. Would you like to ask me any questions?

Thank you a lot for your time and information
